# Geographical variation in diabetes mellitus prevalence rates in Greece

**DOI:** 10.1900/RDS.2023.19.62

**Published:** 2023-06-30

**Authors:** Antigoni Faka, Louzela-Marina Ntafla, Christos Chalkias, Demosthenes B Panagiotakos

**Affiliations:** 1 Department of Geography, School of Environment, Geography and Applied Economics, Harokopio University, Athens, Greece,; 2 National Organization for Health Care Services Provision – EOPYY, Maroussi, Greece; 3 Department of Nutrition and Dietetics, School of Health Science and Education, Harokopio University, Athens, Greece,; 4 Faculty of Health, University of Canberra, Canberra, Australia.

**Keywords:** diabetes, geographic information systems, health geography, spatial epidemiology, Greece

## Abstract

The aim of this study was to investigate the geographical variation of diabetes prevalence in Greece. The database of Diabetes Mellitus Patients Registry of the National Organization for Health Care Services Provision, was used to identify patients of type 1 and type 2 diabetes. Incidence rates were estimated by type of diabetes and sex for each prefecture of Greece and spatial analysis was performed to recognize statistically significant spatial clusters across the country. 424,118 patients of type 1 and type 2 diabetes had been registered in the Diabetes Mellitus Patients Registry. Type 1 diabetes prevalence was 0.24% and type 2 was 3.66%. Mapping diabetes prevalence revealed the highest rates oftype 1 in the Greek islands, whereas the highest rates of type 2 diabetes were identified in northern and eastern prefectures of Greece. Spatial clusters of high values of diabetics were noticed in northwest and northeast Greece, for type 1 and type 2 diabetes respectively. In type 1, men patients prevailed to women in most Greek prefectures, while type 2 men to women ratio highlighted the female predominance in north, central and east Greece. The present study underlines geospatial surveillance as a useful tool by more precisely determining the underlying spatial epidemiology of diabetes.

## 1 Introduction

Diabetes Mellitus (DM) is characterized by elevated levels of blood glucose, affecting the heart, blood vessels, eyes, kidneys and nerves and causing various disabilities that impact significantly on quality of life [1]. Diabetes is one of four priority noncommunicable diseases (NCDs) and among the leading causes of premature mortality [1,2]. It was estimated that in 2019 diabetes and its complications caused 4.2 million deaths worldwide in the 20-79 years age range and almost half of them (46.2%) occurred in people under 60 years of age [3].

Estimates of the past and future burden of diabetes demonstrate the steadily rise of diabetes global prevalence [4-9]. In 2019, 463 million (9.3%) of the worldwide adult population (20-79 years) was estimated to suffer from DM, with the projections increasing the number of patients to 700 million (10.9%) in 2045 [10].

In Europe, the number of adults (20-79 years) with diabetes was estimated at 59.3 million, representing 8.9% of the regional population in this age group, a number which is expected to increase to 9.8% by 2035 and 10.3% by 2045 [3]. Almost 465,000 deaths (8.5% of all-cause mortality) in Europe were attributable to diabetes and 31.4% of them occurred in people under the age of 60 years.

The increasing diabetes prevalence results in higher mortality, reduced quality of life as well as in greater economic burden on healthcare systems [11]. In 2019, the total estimated medical cost of diabetes in Europe was $161.4 billion [3]. In the USA, the diabetesrelated direct health expenditure in 2017 was $237.3 billion and $89.9 billion indirectly lost due to reduced employment, productivity etc. ($19.9 billion of them due to premature death) [12].

In Greece, over the last decades, several epidemiological studies, mostly at regional and local scale [13-20] and two at national level [21-22], have estimated diabetes prevalence in the country. Liatis et al. [21], based on real-world data from the nationwide prescription database, reported the prevalence of drug-prescribed diabetes in Greece was 7.0% (720,764 individuals). According to the International Diabetes Federation estimates for 2019, the prevalence of diabetes in Greek adults (20-79 years) was 7.4% and the diabetes-related deaths was 3,231 [3].

Among other negative impacts of diabetes, and taking into account the annual prescription cost per diabetic patient in Greece was estimated at 1,147€ in 2018 [23], and the 90% to 100% of anti-diabetic medications cost is covered by the National Health System, diabetes also brings about substantial economic loss to the Greek national economy and health system.

The negative impacts of premature mortality and disabilities due to diabetes and its complications lead to the need for more effective policies at reducing diabetes prevalence. Spatial epidemiology and health geography are increasingly being used to investigate the geographical variation of a disease and recognize high-risk areas [24-26], being key factors in targeted interventions, that are efficient and cost-effective. Mapping the disease distribution, recognizing spatial patterns and identifying the main causal factors of the observed patterns, support a more effective decision making for disease intervention [26] resulting in better control of the disease, the reduction of its prevalence, the improvement of public health and the decrease of the total medical care cost.

Geospatial analysis of diabetes has significant role in health research and spatial epidemiology [25,27-37]. Nevertheless, in Greece the research on the geography of diabetes is limited to one study, at local scale [38]. On that account, the aims of this study were to investigate the geographical variation of diabetes prevalence across the Greek prefectures by type of diabetes and sex, identify statistically significant spatial clusters and recognize high-risk areas, based on all patients recorded in the Diabetes Mellitus Patients Registry of the National Organization for Health Care Services Provision (EOPYY), between January 21 and August 22, 2019.

## 2 Materials and Methods

### 2.1 Data

EOPYY is the national health-care provider in Greece. From January 21, 2019, patients of all DM types should be registered in the DM Patients Registry of EOPPY, based on the unique social security number, in order to prescribe diabetes consumables products and the cost to be covered from the National Health System.

**Figure 1.**
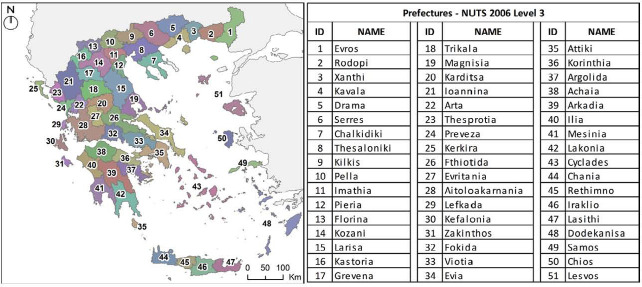
Prefectures of Greece (NUTS 2006 level 3)

Diabetic patients registered to the DM Patients Registry from January 21 to August 22, were obtained from EOPYY. Patients were classified by ICD-10 code (International Statistical Classification of Diseases and Related Health Problems 10^th^ Revision), sex and prefecture (NUTS 2006 level 3, https://eur-lex.europa.eu/legal-content/EN/TXT/ PDF/?uri=CELEX:32007R0105&from=EN) (Figure 1). The classification of the patients in type 1 (T1DM) or type 2 diabetes (T2DM) was based on the ICD-10, version 2019, of the World Health Organization (https://icd.who.int/browse10/2019/en#/E10-E14). Patients of T1DM were identified according to the ICD-10 code for insulin-dependent diabetes (E10) and patients of T2DM based on the code for no-insulin-dependent diabetes (E11). All other cases of ICD-10 codes relevant to diabetes were not included in the analysis.

Diabetes prevalence by sex and prefecture was estimated based on the corresponding characteristics of the total Greek population, according to the 2011 census data of the Hellenic Statistical Authority (https://panorama.statistics.gr/en/) (Figure 1).

### 2.2 Spatial analysis

Geographic Information Systems (GIS) technology was used to map the prevalence of diabetes across Greek prefectures and identify spatial patterns of highrisk areas. A geodatabase was created, including the spatial layers of the Greek prefectures, diabetics data and census data of total Greek population. A series of GIS-supported procedures were implemented to geocode and aggregate all data by spatial unit. Share of patients with T1DM was expressed per 10,000 population and T2DM per 1,000 population.

Hot Spot Analysis was performed implementing Getis-Ord Gi* statistic [39-40] to identify statistically significant spatial clusters of high values (hot spots) and low values (cold spots). The standardized Gi* is essentially a z-value associated with statistical significance (99%, 95% and 90% confidence level), revealing where geographical units with either high or low values cluster spatially.

In Hot Spot Analysis the spatial relationship parameter and the distance method should be defined (https://desktop.arcgis.com/en/arcmap/10.3/tools/spatial-statistics-toolbox/hot-spot-analysis.htm). The spatial relationship parameter was based on the context of neighboring spatial units by defining a critical distance. Spatial units outside the critical distance have no influence on a target unit’s computations. The threshold distance was set by default, which is the minimum distance that ensures each unit has at least one neighbor. As a result, insular prefectures were excluded from spatial patterns analysis. In the distance method, which specifies how distances are calculated from each spatial unit to neighboring units, the Euclidean distance (straight-line distance) was defined.

Spatial analysis enabled the production of a series of thematic maps, illustrating the geographical variation of diabetes prevalence across the country and the statistically significant hot and cold spots. The incidence rates’ values classification was based on the national statistical mean of each rate. This method highlights each prefecture’s state compared with the average country’s state. In the produced maps, five classes were specified according to the country’s mean value (central class) and the respective statistical means of the values below (two lower classes) and the values above (two upper classes) the country’s average. Spatial analysis and mapping were performed using ArcGIS version 10.2 (ESRI Inc., Redlands, California, USA).

## 3 Results

424,118 patients of T1DM and T2DM had been registered in the DM Patients Registry of EOPPY till August 22, 2019. 396,226 patients with T2DM were identified, corresponding to a prevalence of 3.66% and accounted for 93.4% of the two basic types of diabetes. T1DM prevalence was 0.24% (27,892 individuals). Prevalence was almost identical between sexes in both types of diabetes, as there were 194,714 men (3.67%) and 201,512 women (3.66%) with T2DM, and 14,586 men (0.28%) and 13,306 women (0.24%) with T1DM.

**Figure 2.**
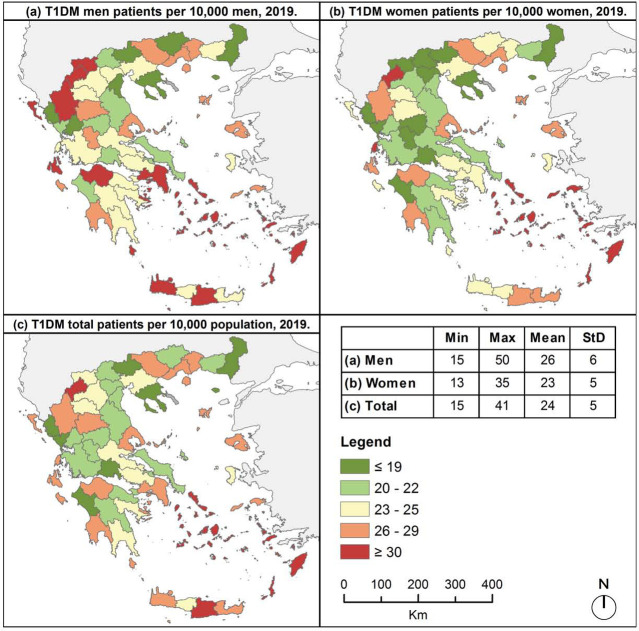
Prevalence of T1DM across Greek prefectures, 2019

Mapping diabetes prevalence across prefectures of Greece revealed high rates of T1DM in almost all insular prefectures, with the highest rates in prefectures of Cyclades, Dodekanisa and Iraklio (Figure 2c). One prefecture in mainland Greece (Kastoria) was also characterized by very high rates of diabetes, whereas the rest mainland prefectures were characterized mostly by low rates. High rates of diabetes were also revealed in insular prefectures in both women and men patients (Figures 2a and 2b). In mainland prefectures men had higher rates than women, especially in Florina, Kastoria, Ioannina, Attiki and Achaia, where men had the highest rates (Figure 2a).

**Figure 3.**
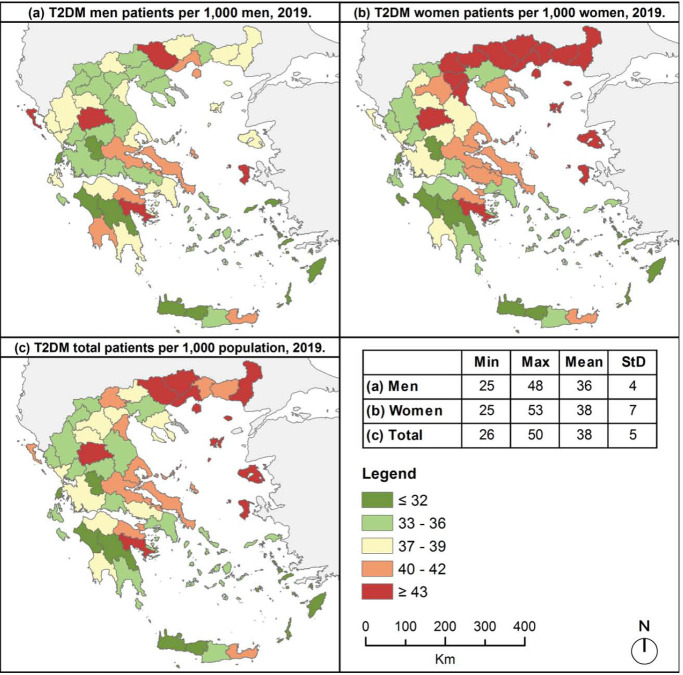
Prevalence of T2DM across Greek prefectures, 2019

On the other hand, insular prefectures were characterized by low rates of T2DM, but the northeastern islands (prefectures of Chios and Lesvos) (Figure 3c). In the mainland Greece, T2DM was most prevalent in northeastern prefectures and the prefectures of Trikala and Argolida, with the latter two and the north prefecture of Serres being characterized by high rates in both men and women (Figures 3a and 3b). Furthermore, very high rates in women were identified in ten neighboring prefectures in northcentral and northeastern (Figure 3b).

**Figure 4.**
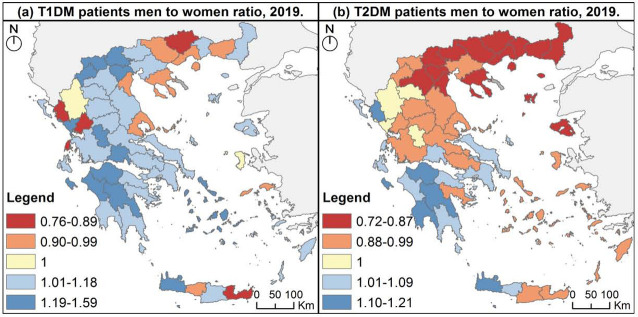
Diabetes patients, men to women ratio, 201

In general, T1DM men patients prevailed to women in most Greek prefectures (Figure 4a), while T2DM men to women ratio highlighted the female predominance in north, central and east Greece (Figure 4b). Nevertheless, men to women ratio was lower than 1 in both types of diabetes mainly in northern Greece, and higher than 1 mostly in southern Greece.

**Figure 5.**
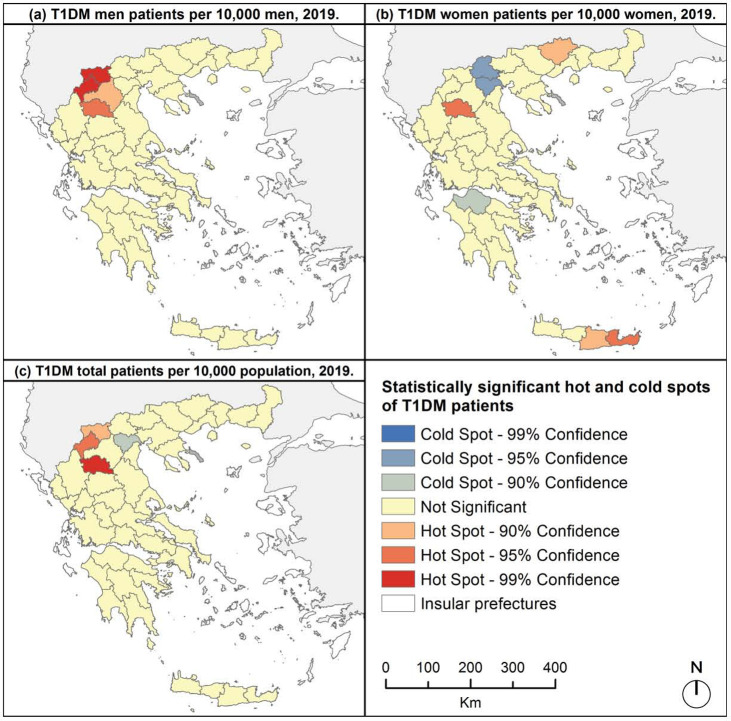
Hot and cold spots of T1DM patients, 2019

Spatial analysis revealed hot spots of T1DM in northwest Greece; in the prefectures of Grevena, Kastoria and Florina, and a spatial cluster of low values in Imathia prefecture (Figure 5c). For men patients, the same hot spots were recognized, plus one in prefecture of Kozani (Figure 5a). Four spatial clusters of high values were also noticed for women patients; in the prefectures of Grevena, Drama, Iraklio and Lasithi (Figure 5b). No statistically significant spatial cluster was observed for men patients, while for women, cold spots were noticed in the prefectures of Pella, Imathia and Achaia (Figures 5a and 5b).

**Figure 6.**
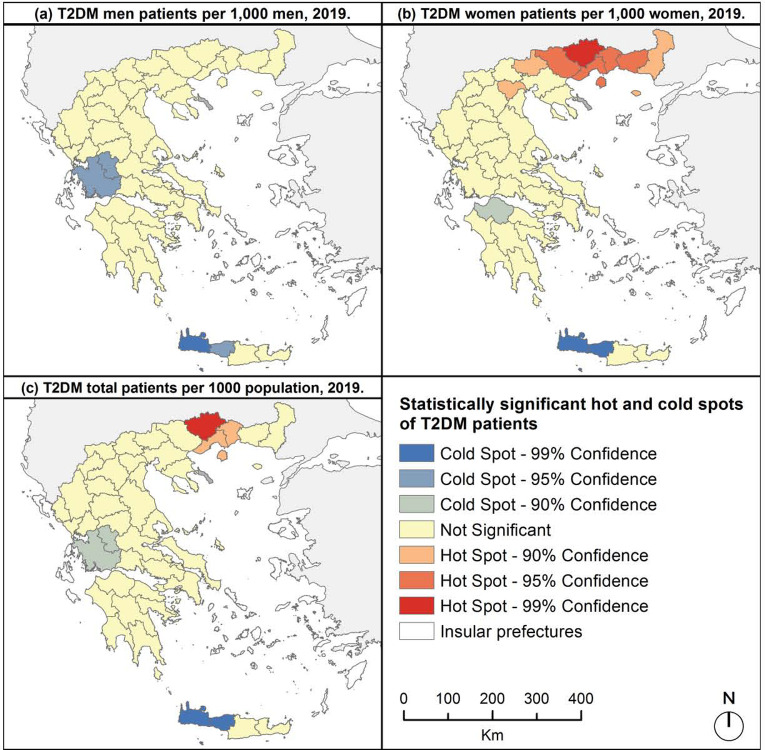
Hot and cold spots of T2DM patients, 2019

For T2DM, spatial clusters of high values were revealed in northeast Greece; in the prefectures of Drama, Kavala and Xanthi (Figure 6c). Cold spots of T2DM were identified in western Crete (prefectures of Chania and Rethimno) as well as in the prefectures of Aitoloakarnania and Evritania. The same cold spots were revealed for men patients, but no hot spot was pointed out (Figure 6a). In western Crete, cold spots were also identified for women patients, plus one in prefecture of Achaia. Hot spot analysis showed significant concentration of high values of T2DM women patients in northeastern Greece, where hot spots were noticed in eight neighboring prefectures (Figure 6b).

## 4 Discussion

This is the first study investigating geographical variation in diabetes prevalence rates and identifying spatial clusters of high/low values across the prefectures of Greece. The analysis was based on real-world data [41] sourced by the Diabetes Mellitus Patients Registry of the National Organization for Health Care Services Provision of Greece.

The study showed that the prevalence of T1DM in the Greek population was 0.26%, in accordance to study estimating T1DM in Greece to 0.24% (Liatis et al., 2016). The prevalence of T2DM was 3.66%, lower than other recent estimates [3,21]. The lower T2DM prevalence is maybe attributed to the fact that not all diabetes patients had been registered in the DM Patients Registry by August 22, 2019. Diabetics are registered to the DM Patients Registry by their physicians by creating an electronic patient folder. Registrations started on January 21, 2019, therefore, patients who had not visited their doctor within these seven months and were not registered, are not included in the dataset.

Overall diabetes prevalence was almost identical between sexes in both types of diabetes, however mapping diabetes prevalence revealed T1DM men patients prevailed to women and T2DM women predominated over men in most Greek prefectures. This outcome is based on the different distribution of men and women population in the Greek prefectures, underlying the evidence that the larger the geographic level of analysis, the greater the potential ignorance of internal heterogeneity.

Mapping diabetes prevalence highlighted the geographical variation of diabetes in Greece. In all insular prefectures, except two, T1DM rates were higher than the country’s mean value. In northeastern islands high incidence rates of T2DM was also identified. In general, the residents of Greek islands are confronted with substantial deficiencies in health care facilities and services [42]. The inequality in access to health services is increased due to the geographic fragmentation of the insular prefectures. Hence, and based on the fact that in many Greek islands there are no specialized diabetes departments, these findings enhance the need for establishing health structures and provision for diabetes care in the islands.

On the other hand, T2DM was most prevalent in mainland Greece, especially in northeastern prefectures, where statistically significant hot spots were also identified. The high concentrations of diabetes patients in this area are maybe attributable to their dietary patterns. The population of northern Greece comprises several ethnic groups, including, among others, Muslim minority (Turks, Pomaks and Romani), Greek Pontians and Asia Minor Greek refugees, whose dietary patterns are characterized by high consumption of starchy foods, red meat (mostly lamb) and highfat dairy. Many studies have reported the positive association of such dietary patterns with diabetes [43,44]. On the contrary, statistically significant cold spots of T2DM were identified in western Crete. The Seven Countries Study attributed the very good health status of Cretan population to dietary habits and the positive health effects of the Mediterranean Diet [45], the high adherence to which is inversely associated with the risk of diabetes development [13,46-49]. Cold spots of T2DM were also revealed in two prefectures in central-western Greece, mostly characterized by rural areas. Prevalence of diabetes is lower in rural than urban areas, as it has been estimated than almost two thirds of diabetes patients live in urban environments [4].

Many studies have already examined diabetes prevalence in Greece, though, this is the first one to document diabetes disparities across the country. Adding geographical dimension and investigating spatial heterogeneity of diabetes is the main strength of this study. GIS-based analysis improves the understanding of diabetes prevalence in a spatial context [25,27,29-30,32,38,50], giving spatial epidemiology a big advantage over other methods. In Greece, there has been developed an insufficient number of clinics relevant to diabetes in public hospitals [51], and this study may contribute to a more effective planning of development of diabetes centers network. Furthermore, this study used real-world data, trying to cover the total Greek population. However, the DM Patients Registry may have not included all diabetics at the time the data were obtained, as EOPYY launched the registry platform seven months ago and the patients should be registered by their doctors. Another limitation is related to undiagnosed diabetes patients, accounting for almost 36% of cases in Greece [3], who are not registered in the database.

It is well accepted that diabetes is a global health challenge and this study used geographic analysis to investigate spatial distribution of diabetes in Greece. Spatial analysis in epidemiology constitutes a powerful tool for health decision-makers. Understanding the geography of diabetes and detecting high diabetes risk areas may support the development of targeted interventions, such as the establishment of specialized diabetes departments in high-risk areas or the enhancement of existing diabetes clinics in public hospitals and the strengthening of diabetes medical care in hot spots. In future work, a study combining diabetes prevalence and the spatial distribution of diabetes-related health structures in Greece, could indicate areas under the need of such interventions. Furthermore, the spatial heterogeneity of diabetes prevalence across the Greek prefectures could be examined by age, to identify differences among age groups. Finally, the spatial analysis could be performed at lower geographical level and over time periods to identify diabetes heterogeneity at local scale and changes over time.

### 4.1 Conclusions

The present study demonstrated that the diabetes prevalence differs significantly across the Greek prefectures. This spatial heterogeneity of diabetes prevalence may reflect differences in local lifestyles, dietary patterns, diabetes prevention awareness and access to health services, since there were higher prevalence rates of diabetes in areas with diabetes associated dietary patterns and deficiencies in health care facilities and services. Mapping diabetes prevalence and identifying statistically significant spatial clusters indicate high diabetes risk areas that constitute a priority objective of diabetes awareness interventions and health structures enhancement.

## Abbreviations

DM: Diabetes Mellitus

EOPYY: National Organization for Health Care Services Provision (of Greece)

GIS: Geographic Information Systems

ICD: International Classification of Diseases

NCDs: Non-Communicable Diseases

NUTS: Nomenclature of Territorial Units for Statistics

T1DM: Type 1 Diabetes Mellitus

T2DM: Type 2 Diabetes Mellitus
